# Late-onset intracranial adenocarcinoma arising from a germ cell tumor 25 years after initial diagnosis

**DOI:** 10.1007/s10014-025-00522-2

**Published:** 2025-11-25

**Authors:** Yuri Yamagiwa, Masashi Nomura, Hirokazu Takami, Atsushi Kondo, Yosuke Kitagawa, Aya Shinozaki-Ushiku, Shunsaku Takayanagi, Nobuhito Saito

**Affiliations:** 1 Department of Neurosurgery, Graduate School of Medicine, The University of Tokyo, 7-3-1 Hongo, Bunkyo-Ku, Tokyo, 113-0022 Japan; 2 Department of Pathology, Graduate School of Medicine, The University of Tokyo, 7-3-1 Hongo, Bunkyo-Ku, Tokyo, 113-0022 Japan; 3 Department of Neurosurgery/Neuro-Oncology, Saitama Medical University International Medical Center, 1397-1 Yamane, Hidaka-Shi, Saitama, 350-1298 Japan

**Keywords:** Germ cell tumor, Teratoma with somatic-type malignancy, DNA methylation

## Abstract

A teratoma with somatic-type malignancy is a rare subtype of germ cell tumor (GCT) characterized by the presence of a histologically distinct component resembling a somatic cancer within a teratoma feature. To date, only 14 cases have been reported in the central nervous system (CNS), and its diagnosis remains challenging due to the limited availability of pathological findings. A case of a pineal GCT that recurred as an adenocarcinoma 25 years after the initial chemoradiotherapy is reported. Although the histological features of teratoma were not observed, transformation into a teratoma with somatic-type malignancy was suspected. DNA methylation analysis classified the tumor within the CNS GCT cluster, specifically aligning with teratomas. Furthermore, whole-exome sequencing demonstrated multiple somatic mutations including two MAPK pathway gene alterations. These findings support the hypothesis that a GCT can evolve into a somatic cancerous phenotype over time, providing deep biological insights into the carcinogenesis of teratomas.

**Supplementary Information:**

The online version contains supplementary material available at 10.1007/s10014-025-00522-2.

## Introduction

Primary intracranial teratoma, a subtype of non-germinomatous germ cell tumor (GCT) [1], is rare, accounting for only 0.3–0.6% of all intracranial tumors. It contains cellular components that recapitulate the differentiation potential of three germ layers: ectoderm, mesoderm, and endoderm. It most commonly occurs along the midline of the brain and exhibits diverse clinical presentations depending on the location.

Teratoma with somatic-type malignancy, previously known as teratoma with malignant transformation, is exceedingly rare, with only 14 cases reported to date [2–9]. A unique case of a pineal GCT that transformed into a teratoma with somatic-type malignancy 25 years after initial treatment is presented. Unlike typical cases, the present case exhibited only an adenocarcinoma phenotype, with no detectable teratoma elements. A diagnosis of teratoma with somatic-type malignancy was made based on pathological examination and DNA methylation analysis.

## Clinical summary

A 38-year-old man presented to our neurosurgery clinic with progressive short-term memory impairment and a writing disturbance. He had a history of treatment for a pineal tumor and obstructive hydrocephalus 25 years earlier. At that time, there was a high degree of suspicion that the pineal lesion was a GCT based on the radiographical findings, and it was treated with chemotherapy and radiotherapy without a histological diagnosis. A ventriculoperitoneal (VP) shunt was placed to manage the hydrocephalus. The pineal lesion subsequently shrank and eventually disappeared in response to treatment. The VP shunt was ligated. There were no clear signs of tumor recurrence on magnetic resonance imaging (MRI) three years earlier.

**Fig. 1:**
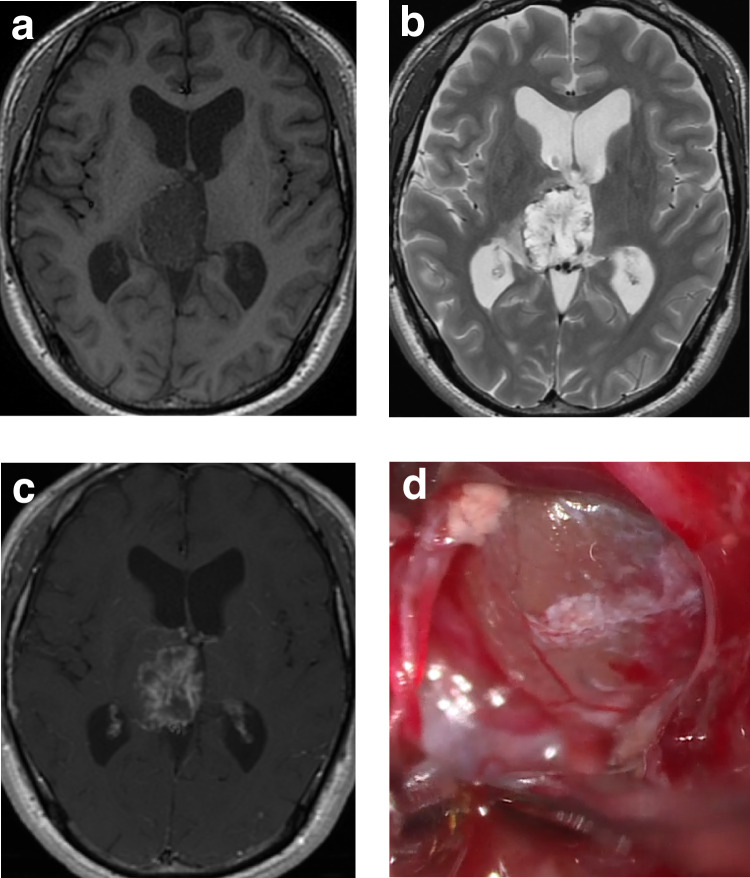
Pre-operative images and intra-operative findings. Magnetic resonance imaging (MRI) of the brain showing a pineal lesion that appears hypointense on a T1-weighted image (**a**) and hyperintense on a T2-weighted image (**b**). The lesion is enhanced on the post-contrast T1-weighted image (**c**). Intraoperative microscopic image in the second surgery shows a grayish tumor with a white membranous structure (**d**)

At this presentation, computed tomography (CT) of the brain showed an approximately 39-mm, well-defined, multilobulated, solid-cystic lesion with calcification in the region of the quadrigeminal cistern and right posterior parasagittal area, accompanied by enlargement of the lateral ventricle. It was considered highly likely that the lesion was a recurrence of the GCT. On MRI, a lesion that appeared hypointense on T1-weighted images and heterogeneously hyperintense on T2-weighted images (Fig. 1a, b) was seen. It was heterogeneously enhanced on the post-gadolinium T1-weighted images (Fig. 1c). The constructive interference in steady state (CISS) images showed obstruction of the aqueduct, likely contributing to the hydrocephalus. Blood tests showed slight elevations in carcinoembryonic antigen (CEA) and carbohydrate antigen 19-9 (CA 19-9) levels, whereas alpha-fetoprotein (AFP) and beta-human chorionic gonadotrophin (β-HCG) levels were within normal limits.

To manage the obstructive hydrocephalus and obtain a histological diagnosis of the tumor, the patient underwent endoscopic surgery. Following third ventriculostomy, the tumor with a grayish, membranous appearance was biopsied.

## Diagnosis based on the pathological and molecular findings

**Fig. 2:**
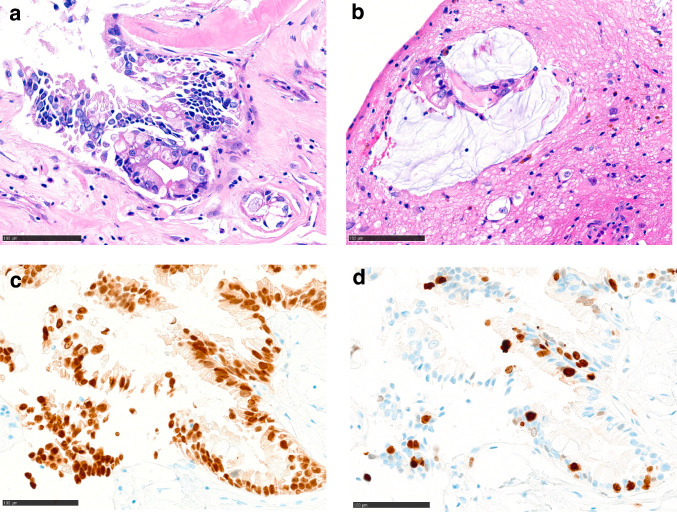
Histological findings of the tumor. Representative histological images of the tumor. The tumor consists of mucin-producing cells showing tubular growth (**a**: HE). The tumor invaded the brain parenchyma with extracellular mucin pool and surrounding gliosis (**b**: HE). Immunohistochemically, the tumor cells are positive for CDX-2 (**c**), and the MIB-1 proliferation index is approximately 10% (**d**)

Histologically, the tumor was composed of atypical epithelial cells with clear to eosinophilic cytoplasm and mucin production, forming tubular and papillary structures (Fig. 2a). The tumor cells exhibited marked nuclear atypia and mitotic figures, and were associated with invasion into the brain parenchyma with abundant extracellular mucin (Fig. 2b). Immunohistochemical staining was positive for CDX-2 (Nichirei, 418,011, EPR2764Y, rabbit, monoclonal, prediluted.) (Fig. 2c) and negative for SALL4 (Santa Cruz, sc-101147, EE-30, rabbit, monoclonal, 1:50.). The MIB-1 proliferation index (Roche, 05278384001, 30-9, mouse, monoclonal, prediluted.) was approximately 10% (Fig. 2d). These features were consistent with an adenocarcinoma exhibiting differentiation toward gastrointestinal-type epithelium, with no clear evidence indicating GCT. Histological examination of the resected tumor confirmed the adenocarcinoma feature, but no teratoma component was identified. To rule out intracranial metastasis from a truncal neoplastic lesion, the patient underwent whole-body contrast-enhanced CT and PET imaging, but no cancerous lesions were detected outside of the brain.

**Fig. 3:**
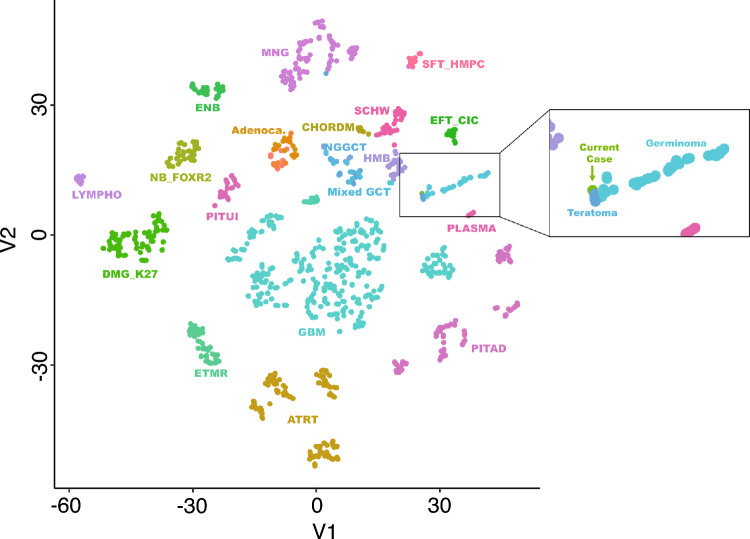
DNA methylation analysis of the tumor shows a shared epigenetic profile with GCTs. The *t*-distributed stochastic neighbor embedding (*t*-SNE) analysis of the DNA methylation array data (EPIC v2.0) performed with public datasets of representative brain tumors (GSE109381, n = 986), intracranial GCTs (GSE70783, n = 83) and 4 types of adenocarcinomas (https://portal.gdc.cancer.gov, n = 40) clusters current case in GCTs, specifically close to teratomas. Adenoca., adenocarcinoma (colon, esophagus, rectum, stomach); ATRT, adult pituitary atypical rhabdoid/teratoid tumor; CHORDM, chordoma; DMG_K27, diffuse midline glioma, H3K27M; ENB, esthesioneuroblastoma, subclass B; EFT_CIC, Ewing sarcoma family tumor with CIC alteration; EWS, Ewing sarcoma; ETMR, embryonal tumor with multilayered rosettes; GBM, glioblastoma; HMB, hemangioblastoma; LYMPHO, lymphoma; Mixed GCT; mixed germ cell tumor; MNG, meningioma; NB_FOXR2, CNS neuroblastoma with FOXR2 activation; NGGCT, non-germinomatous germ cell tumor; PITAD, pituitary adenoma; PITUI, pituicytoma; PLASMA, plasmacytoma; SCHW, schwannoma; SFT_HMPC, solitary fibrous tumor

To clarify the deep molecular underpinnings of the tumor and make a definitive diagnosis, DNA methylation and mutation analyses were performed with the approval of the research ethics committee of the University of Tokyo (G10028) and the patient’s written, informed consent. Genomic DNA was extracted from the frozen tumor tissue and blood sample using DNeasy Blood and Tissue kit (Qiagen 69,504, Tokyo, Japan). The extracted tumor DNA was analyzed by Infinium Methylation EPIC methylation array v2.0 (Illumina, CA, USA), following the manufacturer’s protocol. The raw signal data intensities derived from IDAT data file was processed by minfi Bioconductor package (version 1.44.0, R version 4.2.2) to calculate the β value and used for t-distributed stochastic neighbor embedding (t-SNE) analysis. The analysis was done with public DNA methylation array dataset of representative brain tumors (GSE109381, n = 986), intracranial GCTs (GSE70783, n = 83) and adenocarcinomas (COAD, colon; ESCA, esophagus; READ, rectum; STAD, stomach) randomly selected from in The Cancer Genome Atlas dataset (https://portal.gdc.cancer.gov, n = 10 per each) using the Rtsne package (version 0.15) on the 20,000 most variable probes. On this analysis, the tumor was clustered together with the samples of GCTs, specifically close to the teratoma samples (Fig. 3). The tumor DNA, as well as matched blood DNA, was also analyzed by whole-exome sequencing. Briefly, the DNA was sheared into short fragments (180–280 bp), captured with SureSelect Human All ExonV6 (Agilent, CA, USA), processed with Rapid Plus DNA lib Prep Kit for Illumina V2 (Illumina, CA, USA), and sequenced with 150-bp, paired-end sequencing on a NovaSeq 6000 (Illumina, CA, USA). Tumor purity inferred by ABSOLUTE was 0.5. A mutation call was done with GATK and Mutect, identifying 69 non-synonymous mutations in coding sequencing, which included multiple MAPK signaling pathway mutations (*RAF1*, and *MAP2K6*) (Supplementary Table S1). Copy number variation (CNV) analysis was performed by Control-FREEC v11.4 with the default parameters [10] and revealed partial chromosomal losses and gains (Supplementary Table S2).

## Discussion

In this case, 25 years after the initial chemoradiation therapy for a presumptive pineal GCT and its complete response, a large tumor appeared in the same region, which was suspected to be a recurrence of the initial GCT. However, pathological examination of both the biopsy and the resected tumor showed histological features consistent with enteric adenocarcinoma rather than a typical GCT. Despite the absence of teratomatous components, the patient’s clinical course suggested a diagnosis of teratoma with somatic-type malignancy rather than intracranial metastasis of an adenocarcinoma originating from another organ, which was supported by DNA methylation analysis.

Teratoma with somatic-type malignancy, previously known as “teratoma with malignant transformation,” is a rare subtype of GCT, especially in the CNS. It accounted for 2.6% (4/153) of all CNS GCTs in a large cohort from Matsutani et al. [3], and 0.53% (1/190) in a report from the Intracranial Germ Cell Tumor Genome Analysis (iCGT) consortium [9]. A review of the literature identified 14 previously reported cases of teratoma with somatic-type malignancy in the CNS (Table 1) [2–9]. Of 14 cases whose locations were clear, 11 were in the pineal region, 2 in the ventricle, and 1 in the neurohypophysis [2–9]. A teratoma with somatic malignancy was seen at recurrence in 6 cases and at diagnosis in 3 cases. The malignant components in these cases varied, including adenocarcinoma (4 cases), epidermal carcinoma (4 cases), and embryonal sarcoma (2 cases). The characteristic of teratoma with somatic-type malignancy and immature teratoma is marked enhancement in the solid portion of the lesion, whereas there is moderate, heterogeneous enhancement in the solid portion of the lesion in mature teratoma [11]. To our knowledge, there have been no prior reports of a teratoma with somatic type malignancy presenting as a recurrent lesion so many years after the initial treatment of GCT. We believe that our report contributes to a better understanding of the clinical features of this entity.

**Table 1:** Summary of previously reported cases of teratoma with somatic-type malignancy

Case	Study	Age/sex	Location	Primary lesion or recurrence	Preoperative therapy and surgical resection	Postoperative treatment	Histopathology	Status from the diagnostic
1–2	Freilich et al. [2]	Case 1 26/M	pineal legion	primary lesion	subtotal resection	radiotherapy	yolk sac tumor with adenocarcinomatous transformation of teratoma	recurrence after 6 months
		Case 2 8/M	pineal legion	recurrence	CD VP shunt (no tumar resection)^®^ subtotal resection	CD radiotherapy^®^ radiotherapy	CD yolk sac tumar^®^ enteric-type adenocarcinomatous transformation of teratoma	recurrence after 6 months
3–6	Matsutani et a1. [3]	Case 3–6 31 ± 14.6/M	pineal lesion (1), neurohypophysis (1), other lesions (2)	details unknown	subtotal resection	none/radiotherapy/chemoradiotherapy (details unknown)	epidermal carcinoma (3), sarcoma	10-year survival rates: 70.7%
7	Diyora et a1. [4]	Case 7 50/M	pineal legion	primary lesion	gross total resection	radiotherapy	adenocarcinomatous transformation of mature teratoma	no recurrence (details unknown)
8	Satake et al. [5]	Case 8 14/M	pineal legion	primary lesion (after chemotherapy)	gross total resection	chemoradiotherapy	teratoma with malignant features	relapse-free for over three years
9–11	Faure Canter et al. [6]	Case 9 1.5/M	pineal legion	recurrence	details unknown	chemoradiotherapy	CD immature teratoma^®^ pinealoblastoma	death after 14 months
		Case 10 2.5/F	pineal legion	recurrence	details unknown	chemoradiotherapy	CD immature teratoma^®^ embryonal rhabdomyosarcoma	death after 11 months
		Case 11 9/F	pineal legion	recurrence	details unknown	chemoradiotherapy	CD hCG secreting tumor^®^ pinealoblastoma	death after 24 months
12	Kim et al. [8]	Case 12 54/M	pineal legion	recurrence	CD gross total resection^®^ gross total resection	not mentioned	CD mature cystic teratoma^®^ adenocarcinoma developing in the recurrent intracranial mature cystic teratoma	not mentioned
13	Kamiya et al. [7]	Case 13 16/M	CD pineal legion^®^ septum pellucidum	recurrence	CD VP shunt + radiotherapy- > gross total resection^®^ whole brain radiation therapy-gross total resection	CD none^®^ none	CD mature teratoma^®^ teratocarcinoma (epithelial component was regarded to be malignant)	not mentioned
14	Takami et al. [9]	Case 14 details unknown	unknown	details unknown	details unknown	chemoradiotherapy	teratoma with somatic type malignancy (rhabdomyosarcoma)	intermediate prognosis (details unknown)
15	Our case	Case 15 38/M	(IXg) pineal lesion	recurrence	CD VP shunt (no tumor resection)^®^ subtotal resection	CD chemoradiotherapy^®^ radiotherapy	CD details unknown^®^ adenocarcinomatous transformation of mature teratoma	no recurrence at 9 months

① primary lesion; ②, ③ recurrent lesion

In this report, we applied DNA methylation profiling and whole-exome sequencing to support the diagnosis of teratoma with somatic type malignancy. DNA methylation is a stable epigenetic marker that reflects cellular origin and has proven useful in classifying central nervous system (CNS) tumors in clinical practice [12]. While the present tumor showed features of a fully differentiated adenocarcinoma, DNA methylation analysis demonstrated a profile closely resembling that of a GCT, particularly teratoma, suggesting that this lesion likely arose from a teratoma with subsequent differentiation into a somatic-type malignancy. This highlights the powerful role of DNA methylation profiling in diagnostic clarification. We also performed whole-exome sequencing on this tumor, as the genomic features of teratoma with somatic-type malignancy remain unclear. In the present case, whole-exome sequencing showed a relatively high mutation burden (69 non-synonymous mutations) compared with a previous cohort of 41 CNS GCTs (mean = 16.1, median = 8.0) analyzed by Ichimura et al. using whole-exome sequencing [13]. The present analysis identified 2 mutations in the MAPK pathway, aligning with the key genomic characteristics of the CNS GCTs shown in the previous study. Future studies are needed to identify driver genomic events underlying phenotypic transformation.

The longitudinal evolution of histopathological features in GCTs is poorly understood. A recent long-term, follow-up study of 228 CNS GCTs (median follow-up period 222 months) demonstrated that recurrence can occur even decades later, with up to 29% of germinomas recurring, underscoring the importance of long-term follow-up [14]. Notably, of 7 initial germinomas with available recurrent specimens, 2 recurred as non-germinomatous GCTs, suggesting that GCT evolves to an aggressive phenotype over time. In the present case, given the good response to initial chemoradiotherapy and the prolonged interval before recurrence, the initial tumor was likely a GCT subtype with a good clinical course, such as germinoma. The mechanism underlining the transformation remains unclear. One hypothesis is that the residual tumor cells surviving chemoradiotherapy differentiated into the teratomatous cells, which subsequently transformed into adenocarcinomatous cells. Alternatively, the teratomatous component may have been present from the outset and underwent malignant transformation after prolonged dormancy. These two hypotheses have also been previously proposed by Morinaga et al. in relation to a mediastinal GCT case they reported [15]. However, a critical limitation to consider in the pathomechanism of the present case is the lack of histological information about the first tumor. To clarify the evolutionary mechanisms of GCTs, detailed analysis of the clinical, pathological, and molecular characteristics of further similar cases will be needed.

In summary, this case highlights three important aspects: the exceptional rarity of teratoma with somatic-type malignancy in the central nervous system, the diagnostic power of DNA methylation profiling, and the potential to gain new insights into the mechanisms of pathological transformation in GCTs. Together, these findings strengthen the clinical and biological significance of our report.

## Supplementary Information

Below is the link to the electronic supplementary material.

Supplementary file1 (XLSX 17 KB)

## Data Availability

The data supporting the findings of this study are not publicly available due to privacy concerns. These are available from the corresponding author on reasonable request.
